# India’s Poshan Tracker: data-driven tool for maternal and child nutrition

**DOI:** 10.1016/j.lansea.2024.100381

**Published:** 2024-03-11

**Authors:** Lindsay M. Jaacks, Ananya Awasthi, Apoorva Kalra

**Affiliations:** a Global Academy of Agriculture and Food Systems, The University of Edinburgh, Midlothian, UK; b Anuvaad Solutions, New Delhi, India

Early detection and intervention are key for addressing undernutrition in children.^1^ Increased global coverage of mobile phones could be a game-changer for nutrition surveillance. By reducing the cost and increasing the coverage and speed of nutrition information systems, technology-enabled solutions hold the promise of transforming data-informed decision making. It also has the potential to improve the accuracy of nutrition monitoring data by automating complex calculations, such as the calculation of z-scores from the WHO Growth Tables.

The Government of India’s ‘Poshan Tracker’ is the largest mobile phone-based nutrition surveillance system in the world. It provides transparent data on anthropometric outcomes, functioning of Anganwadi Centres (AWCs) and receipt of care services, namely the provision of supplementary food to women, children and adolescent girls. AWCs are centres established as part of the Integrated Child Development Services Scheme, a flagship government programme aimed at improving the nutritional and health outcomes of mothers and children. We analysed Poshan Tracker data to understand how this innovation in accountability may be influencing real-time monitoring of target beneficiaries and the provision of nutritional services in India. Comprehensive data are publicly available from July 2022 to September 2023 across all states and union territories from AWCs.

Over the past 15 months, the proportion of the country’s more than 1.39 million AWCs that are open for at least 15 days a month has increased dramatically: from only 35% in July 2022 to 89% in September 2023. Likewise, the proportion open for at least 21 days a month has increased from 24% to 78%. This may be partially attributable to improved reporting by AWC workers.

One of the key nutritional services provided by AWCs is the provision of supplementary food, given that food distribution programmes to pregnant women have been shown to lower the risk of stunting and wasting in infants.^2^ All pregnant women and lactating mothers, children aged 6 months to 3 years, all children with severe acute malnutrition, and adolescent girls aged 14–18 years in north-eastern states and aspirational districts are entitled to receive supplementary food.^3^ Supplementary food includes take home rations, which are distinct from raw rations provided through other government services such as the public distribution system. In September 2023, 20.9 million beneficiaries received take home rations for at least 21 days. This is in comparison to 4.1 million in July 2022.

In addition to providing supplemental nutrition, AWCs are the focal point for child growth monitoring in India. In September 2023, 83.55 million children aged 0–6 years had their height and weight measured, representing 94% of children registered at AWCs. This represents a substantial increase since July 2022, when 40.1 million (45%) children aged 0–6 years were measured. As with other indicators, the increase could partially reflect improved reporting. Looking at the most recent national data from September 2023, the Poshan Tracker indicated that 39% of children beneficiaries aged 0–6 years experienced stunting and 18% were underweight. Among children 0–5 years, 6% experienced wasting and 6% were overweight.

The latest population-based anthropometric data for children in India, the National Family Health Survey (NFHS-5, 2019–2021), measured children under 5 years. To improve comparability to Poshan Tracker, we restricted our analysis to the subpopulation of NFHS-5 children who reported being measured at least once in the past year at an AWC (56% of the sample of children). Results indicate that the Poshan Tracker often estimates a lower prevalence of undernutrition than the subset of children in NFHS-5 who report having their weight measured at an AWC in the past year. On average, stunting was estimated to be 1.86 percentage points lower in Poshan Tracker data than NFHS-5; underweight was 13.72 percentage points lower; and wasting was 12.16 percentage points lower. Overweight, in contrast, was 0.68 percentage points higher in Poshan Tracker, on average, than in NFHS-5. There was substantial variability amongst states, and this variability was not consistent across measures (Fig. 1). For example, in Maharashtra, the prevalence of stunting was 9.34 percentage points *higher* in the Poshan Tracker data than NFHS-5, but the prevalence of wasting was 19.27 percentage points *lower*.

**Fig. 1:**
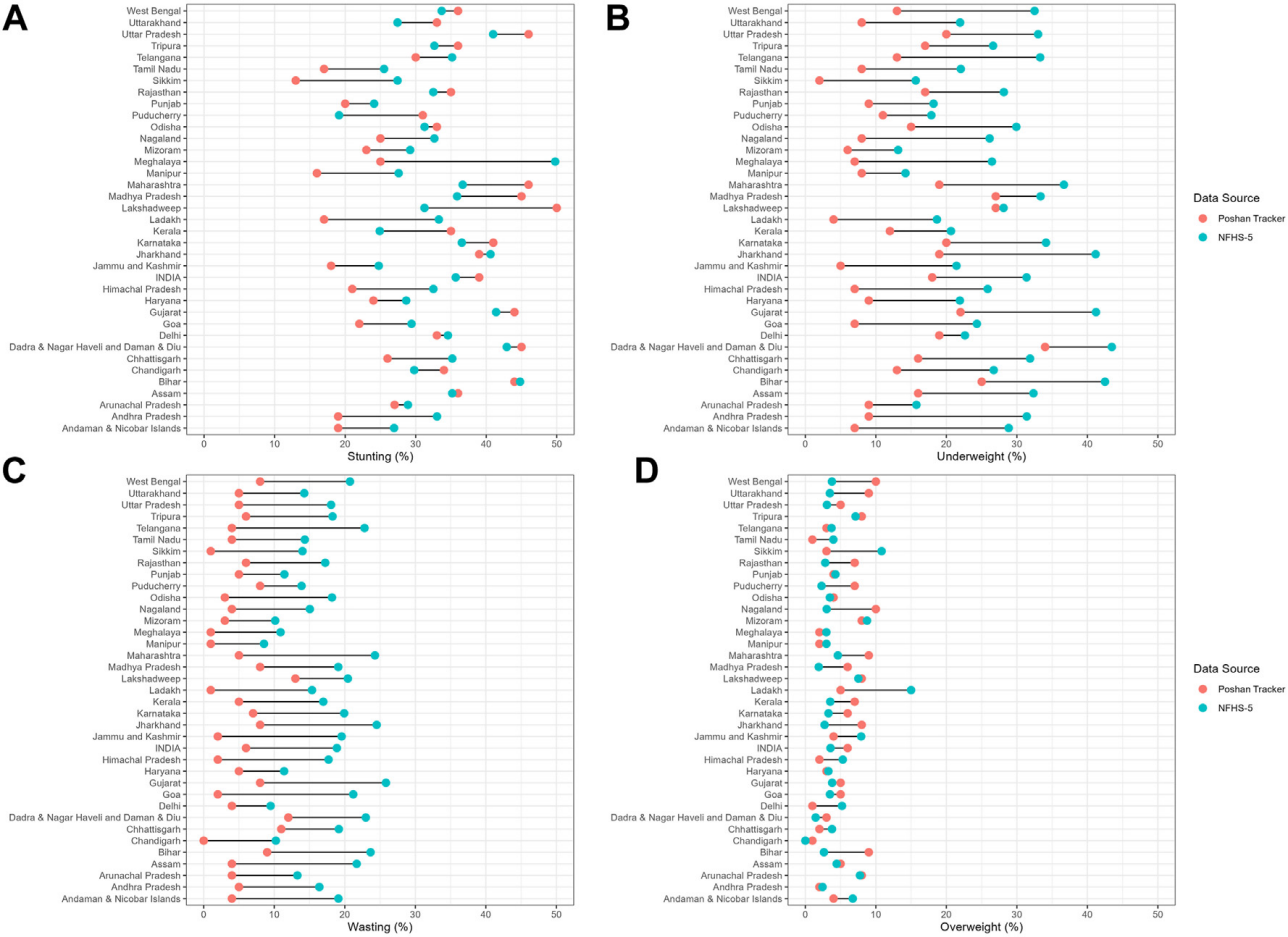
Comparison of (A) stunting, (B) underweight, (C) wasting, and (D) overweight among children under 5 years of age with the exception of stunting and underweight, which are children under 6 years of age for the Poshan Tracker (September 2023). NFHS-5, National Family Health Survey 5 (2019–2021).

These differences could be attributable to a combination of factors. The true prevalence of undernutrition may differ between 2019 and 2021 (NFHS-5) and 2023 (Poshan Tracker). It is possible, for example, that real-time community-based monitoring using the Poshan Tracker has resulted in improved delivery of intervention services, though further evaluations are needed. Future rounds of NFHS will overlap with ongoing Poshan Tracker monitoring and facilitate a more accurate comparison. The training of data collectors and instruments used differs between NFHS-5 and Poshan Tracker. For example, NFHS-5 uses a SECA digital scale whereas most AWCs use spring scales. Moreover, there may be a bias in reporting for Poshan Tracker as data are collected by workers who are tasked with addressing undernutrition in their communities. The data suggest this may be especially true for child weight, which was systematically higher in the Poshan Tracker than in NFHS-5 across all states and union territories, whereas height was higher in some and lower in others.

While not a replacement for routine population-based surveys, real-time community-based monitoring such as the Poshan Tracker can provide a snapshot of the current situation thus allowing for timely identification of areas that require improvement and the potential need to adjust programmatic action to achieve the stated targets. Such systems increase accountability and help ensure that nutrition interventions reach the last mile. Future work should test the pathways by which Poshan Tracker impacts nutritional outcomes. The Poshan Tracker therefore represents an unprecedented opportunity to evaluate the transformative potential of large-scale real-time nutrition monitoring.

## Contributors

LMJ and AA conceptualised the study. LMJ conducted the analysis. LMJ, AK and AA accessed and verified the underlying data reported in the manuscript. LMJ wrote the first draft with critical inputs from AA and AK. All authors reviewed and approved the final manuscript.

## Data sharing statement

All analysis codes are available on GitHub: https://github.com/lindsayjaacks/Poshan-Tracker.

Poshan Tracker data for the current month are freely available from the Poshan Tracker dashboard for those based in India: https://www.poshantracker.in/

We have uploaded monthly all-India and state-wise data on GitHub: https://github.com/lindsayjaacks/Poshan-Tracker.

National Family Health Survey 5 (2019–2021) data are freely available for download from the Demographic and Health Survey Program website: https://www.dhsprogram.com/data/dataset/India_Standard-DHS_2020.cfm?flag=0.

## Declaration of interests

The authors declare that the research was conducted in the absence of any commercial or financial relationships that could be construed as a potential conflict of interest.
